# Practice of Medicine

**Published:** 1859-07

**Authors:** 


### PRACTICE OF MEDICINE.

THE MANAGEMENT OF THE SHOULDERS IN EXAMINATIONS OF THE CHEST. By *J. W. Corson, M.D.* From this interesting paper we extract the summary, as it embodies all the practical facts contained in it:—

1. That remembering the great value of many reputed "little things" in the science of saving life, and that the chests of *lean* persons give clearest sounds, and are best marked, we may seize this hint from nature, and increase the "physical signs," by either lessening or removing more especially those principal natural obstacles, the great pectorals in front, and the two scapulæ and their muscles behind.

2. This may be effected by using the arms as levers, and the hands as hooks to pull. The process, in each case, involves three principles—*thinning*, *condensing*, and *tightening*. It is illustrated by the simple experiment of placing one forearm of a muscular man behind his back, while the other hangs loosely by his side, when the sound, especially of percussion, will be found heightened below the clavicle of the stretched side in front.

3. That the suggestions here offered are not fanciful theories, but the results of practical observations on several hundred patients, in private and in two large dispensaries, during the past year. The drawings, too, were copied from nature. To throw back the shoulders, and bare the whole front, we need the "*first position.*" It is a repetition of the above experiment with *both* arms. *The left wrist is simply held easily with the right hand behind the loins.* This has many little advantages in obscure cases. It gives symmetry, gets rid of the arms, and fits the coat of flesh closely, like a bandage, for "inspection," makes it tense to increase the resonance of delicate percussion, and *conducts* better the sounds within. It thus aids in distinguishing the more difficult cases of tubercles, pleurisy, pneumonia, or aneurism.

4. That the "*second position*" is the common one of locking the hands over the head to examine the axillæ, and is mentioned to avoid omission. The "*third position*" crosses the arms at the back of the head, with the hands grasping near the elbows, so as to *hoist* the shoulder-blades high up behind, and *thin* the muscles, to search for obscure or limited pleurisy or pneumonia low down near the diaphragm posteriorly.

5. It is very important early, in suspicious cases of cough, to examine carefully the *tops of the lungs behind.* For without any distinct signs in front, consumption, often thus mistaken for a mere throat affection, begins here. A few scattered tubercles are apt to *burrow*, as it were, beneath the top of the shoulder. Here we need the "*fourth position.*" For this the patient crosses arms in front, slightly stooping, *hooks* the hands at the loins, or false ribs, and then stretching upward, he holds fast to increase the tension. The physician aids from behind, by pressing down firmly the shoulders. They are thus *slid off*, the muscles are smoothed down, and the ear, coming closer upon the top of the lung, hears better the sounds.

6. *As worth more than all the rest*, we commend the "*fifth position*," for by natural machinery it wrenches the shoulders forward *out of their beds*, and widely severs them in the rear. In thin persons it often thus stretches out their intervening muscles till like stout broadcloth, and thus quite uncovers the inner and upper part of the lungs behind. To accomplish this, the patient crosses his arms in front, with the stronger outside, grasps with the opposite hands the *two shoulder-joints,* pulls both strongly, and holds fast, to keep them tense. The physician aids to fix the shoulder-blades widely apart at the back by firmly pushing. Even in health, as any one can prove, the soft breathing murmur at the former place of the scapula can be thus nearly doubled. In tubercles it here opens a new field for *palpation,* and especially for *percussion.* It intensifies harsh respiration, or "fatty crackling." In pneumonia, it exaggerates the clear, barrel-like echo of "bronchophony," and in pleurisy, that line between wind and water, the trembling "egophony." It brings out a delicate *new sign,* we have discovered, in bronchitis. It is a kind of prolonged liquid breathing, as if through a layer of wet sponge, heard before or after mucous râles, which we venture to name *moist respiration.*

7. Another new and really useful "physical sign" we have to communicate, is *the comparative stiffness of the shoulder over the lung most diseased, in strong breathing, seen and felt from behind.* For this we may use the "*sixth position.*" Facing the back of patient, a yard distant, near a window or white wall, you tell him to drop his arms, let them hang easily by the sides, "as if dead," and then breathe deeply for a few moments, "like a man a little out of breath." You now "take aim," like a rifleman, across the tops of the shoulders, and then shut your eyes and *feel* them gently swell. Drawing nearer, you notice that the "inferior angles" of the scapulæ move gently in breathing, like the fins of a fish. You can both *see* and *feel* this movement. This *stiffness* of the shoulder in breathing may be decided or slight, local or general. When most at the top, we term it, for convenience, "acromial," and when most at the lower extremity, or inferior angle, we call it "angular." Curiously enough, these last features seem to depend on the higher or lower location of the disease, which thus, as it were, *paralyzes the parts nearest.* An elegant way of testing "angular stiffness," even in a lady fully clad, is to place your two index fingers on the lower points of her shoulder-blades, and watch and feel their movement as she sighs. The causes of this stiffness are supposed to be *loss of upward expansion* in the lung, tenderness, pleuritic adhesions, and weight of morbid deposits. A table of eighteen cases is added, illustrative of this sign. It was least in recent attacks; varied most in phthisis; was slightest in pneumonia, and greatest in chronic pleurisy.

8. A statement of measurements of ten males shows the gain in inches and decimals, by "third," "fourth," or "fifth" positions respectively, between the inferior angles of the scapulæ and the lowest lumbar vertebra, the "superior angles" and the vertebra prominens of the neck, and between the two upper and two lower angles of the scapulæ. Of the whole of the six positions, the first, fourth, *fifth,* and sixth, are the most frequently useful. The others apply to particular cases. Taking into account the pulmonary complications of other diseases, as well as the range of "chest disease," it is believed these various improvements, slight as they seem in detail, really throw light, perhaps, upon many forms of *one-third of the fatal maladies of the race.*

9. On account of its fearful importance, it is hoped they will mainly benefit *tubercular consumption.* Tracing faithfully, by various "marks" and the unhealthy habits of the patient, the agencies leading to the two prevailing causes, *feeble organization,* and *depraved nutrition*—by prompt reform of abuses, generous *animal food,* and free exercise in the *open air,* with tonics, and *cod-liver oil* —we may do much to arrest the disease. *Occasionally we may cure.* The encouraging researches of Hughes Bennett and Messrs. Rogée and Boudet, show, that from the numerous chalky concretions, puckerings, and cicatrices, found at the tops of the lungs in very aged persons, it is probable that *about one-half have recovered* from more or less tubercular deposits during their lives. Four living cases from several others are reported by the writer, of arrest or cure of phthisis of several years' standing. The great question of this paper then is, *What may be the result of average notice, say three months sooner?* Time only can tell. Each physician who reads this is earnestly requested to aid by a faithful trial of this system of examinations in at least *three suitable cases.* The malady is still widely and deplorably fatal. From extensive trial, we firmly believe that, simple as they may seem, this *management of the shoulders,* these expedients for *thinning, condensing,* and *tightening the fleshy walls of the chest, add fully one-third to our power of detecting the earliest signs of consumption.* —(*New York Journal of Medicine,* March, 1859, p. 185.)

